# A New Mechanistic Model for Viral Cross Protection and Superinfection Exclusion

**DOI:** 10.3389/fpls.2018.00040

**Published:** 2018-01-25

**Authors:** Xiao-Feng Zhang, Shaoyan Zhang, Qin Guo, Rong Sun, Taiyun Wei, Feng Qu

**Affiliations:** ^1^State Key Laboratory of Ecological Pest Control for Fujian and Taiwan Crops, Fujian Key Laboratory of Plant Virology, Institute of Plant Virology, Fujian Agriculture and Forestry University, Fuzhou, China; ^2^Department of Plant Pathology, Ohio Agricultural Research and Development Center, Ohio State University, Wooster, OH, United States

**Keywords:** cross protection, superinfection exclusion, turnip crinkle virus, p28, protein polymerization

## Abstract

Plants pre-infected with a mild variant of a virus frequently become protected against more severe variants of the same virus through the cross protection phenomenon first discovered in 1929. Despite its widespread use in managing important plant virus diseases, the mechanism of cross protection remains poorly understood. Recent investigations in our labs, by analyzing the whole-plant dynamics of a turnip crinkle virus (TCV) population, coupled with cell biological interrogation of individual TCV variants, revealed possible novel mechanisms for cross protection and the closely related process of superinfection exclusion (SIE). Our new mechanistic model postulates that, for RNA viruses like TCV, SIE manifests a viral function that denies progeny viruses the chance of re-replicating their genomes in the cells of their “parents,” and it collaterally targets highly homologous superinfecting viruses that are indistinguishable from progeny viruses. We further propose that SIE may be evolutionarily selected to maintain an optimal error frequency in progeny genomes. Although primarily based on observations made with TCV, this new model could be broadly applicable to other viruses as it provides a molecular basis for maintaining virus genome fidelity in the face of the error-prone nature of virus replication process.

“What I am advocating is a point of view, a way of looking at familiar facts and ideas, and a way of asking new questions about them”

– Richard Dawkins, The Extended Phenotype.

## Introduction

Host plants pre-infected with a mild isolate of a virus frequently become protected against secondary infections (superinfections) by more severe isolates of the same virus, or closely related viruses, but remain susceptible to more distantly related viruses. This highly specific self-discriminatory phenomenon, referred to as cross protection, was first documented almost 90 years ago, and has since been used to successfully manage a number of virus diseases of crop plants (Sherwood and Fulton, 1982; Gonsalves, 1998; Goregaoker et al., 2000; reviewed by Ziebell and Carr, 2010; Folimonova, 2013). At the molecular level, the genomes of superinfecting viruses are all but undetectable in most of the cross-protected plants, suggesting a failure of cellular entry or multiplication by the superinfectors (e.g., Chewachong et al., 2015). While a unified mechanistic understanding of cross protection remains elusive, it is important to note that similar superinfection exclusion (SIE) effect can be induced by symptomatic – in place of mild – virus isolates as well, as long as the primary and superinfecting viruses are genetically closely related. Indeed, SIE is most robust when the two are nearly identical – distinguishable only through minimal modifications permitting their differentiation (Li and Roossinck, 2004; Takahashi et al., 2007; Miyashita and Kishino, 2010; Folimonova, 2012; Tatineni and French, 2016; Zhang et al., 2017).

Several recent reviews have masterfully chronicled the historical perspectives, and the evolving views of mechanisms of cross protection and SIE (Ziebell and Carr, 2010; Folimonova, 2013). These reviews provide an excellent foundation on which the current update is built. Therefore, instead of trying to be comprehensive, we will focus our discussions on several recent publications that are beginning to unravel novel mechanistic insights. We hereby wish to apologize to colleagues whose contributions are inadvertently missed as a result of this narrow focus.

Our goal is to advocate an unorthodox explanation to cross protection, SIE, and other related observations by proposing a new model that invokes SIE as an evolutionarily conserved mechanism for maintaining viral genome integrity. While this model could have broader implications on many viruses, our discussions will center primarily on a few plant-infecting, positive sense (+) RNA viruses that were subjects of extensive SIE investigations. We wish to emphatically acknowledge that, due to paucity of relevant research, we have to make some assumptions in the discussions. Since among the purposes of our update are to stimulate debates and to inspire further investigations, we would feel honored if our review stirs up some heated arguments, if only they lead to insightful suggestions on how to best test these assumptions and predictions.

## Does SIE Operate At the Cellular or Whole Plant Level?

Although first discovered in plant virus infections, SIE is known to occur in animal virus infections as well. Investigations of animal viral SIE were mostly carried out in cultured cells, showing that SIE could interfere with the cellular entry by superinfectors, and/or replication of superinfector genomes (Schaller et al., 2007; Tscherne et al., 2007, 2008; Zou et al., 2009; Webster et al., 2013). Whether a non-cell-autonomous phase of SIE exists in animal virus infections is difficult to discern as its manifestation is complicated by the actions of both innate and adaptive antiviral immunities.

Experiments with the plant-infecting citrus tristeza virus (CTV) suggest that SIE operates at two different levels (Bergua et al., 2014). The p33 protein encoded by CTV was found to be required for this virus to elicit SIE to a closely related superinfecting isolate at the whole plant level (Folimonova, 2012), yet dispensable for cellular level SIE, thus identifying two phases of SIE (Bergua et al., 2014). However, note that in both of these studies a CTV mutant lacking p33 coding capacity was used as the primary virus to induce SIE, and a p33-encoding CTV as the superinfector. This set-up makes it difficult to rule out the possibility that the p33-lacking CTV mutant might be partially defective in either cell-to-cell or systemic spread, leaving pockets of cells in infected tissues uninvaded by the mutant virus. These virus-free pockets could then permit the entry and multiplication of the superinfecting CTV, making these cells appear to be SIE-defective, but in fact were unprimed for cellular level of SIE. If this possibility stands, it would suggest that CTV SIE acted primarily at the cellular level. Consistent with this possibility, more recent reports from Dr. Folimonova’s lab found that p33 possesses cell-to-cell movement function (Kang et al., 2015, 2017), and p33 alone expressed from a heterologous CTV was insufficient to elicit SIE (Atallah et al., 2016).

That SIE operates primarily at the cellular level is also strongly corroborated by studies with other plant viruses. Indeed, in many cases the “primary virus” and “superinfector” were delivered to plants simultaneously, yet they still segregated as discrete cell clusters adjacent to each other that replicated one or the other, but not both (Takahashi et al., 2007; Miyashita and Kishino, 2010; Julve et al., 2013; Tatineni and French, 2016; Zhang et al., 2017). That complete establishment of the whole plant “cross protection” requires a substantial head start by the primary virus is not inconsistent with SIE operating at the cellular level. Rather, it suggests that most of the susceptible cells must be pre-occupied by the primary virus in order to block the invasion of the superinfector (Chewachong et al., 2015; Zhang et al., 2015). Furthermore, once the cross protection is established, the superinfector genome could not be detected despite repeated attempts over many weeks (Chewachong et al., 2015). Together these studies suggest that the non-cell-autonomous SIE plays a minimal role in SIE manifestation. Instead, individual virus-infected cells are the primary sites of SIE.

## How Does SIE Selectively Block Highly Similar Viruses?

**FIGURE 1:**
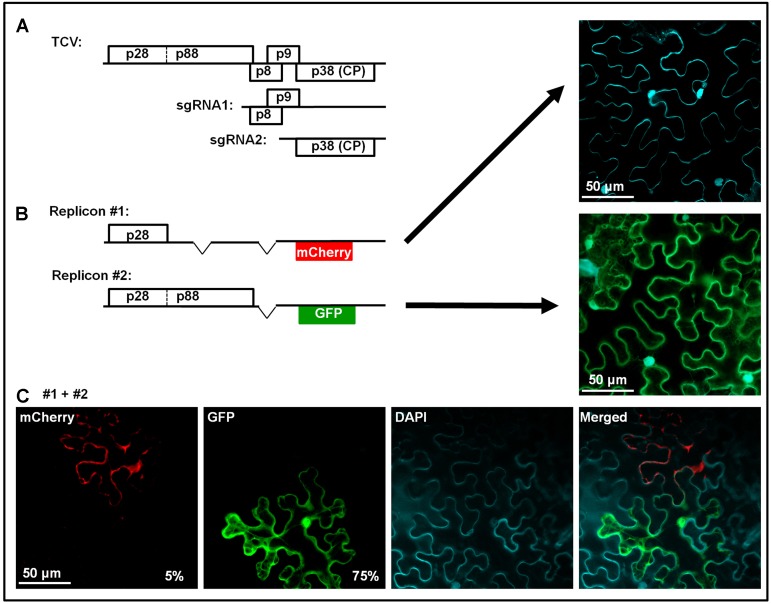
Genome organization of TCV, and dominant SIE exerted by p28. This figure contains previously published data from our lab (Zhang et al., 2017). **(A)** Schematic representation of TCV genomic and subgenomic RNAs, and the proteins encoded. **(B)** Two mutant TCV replicons used in co-infections in **(C)**. Replicon #1 contains deletions within p88 and p8/p9 coding regions, and replacement of p38 by mCherry, thus cannot replicate on its own (top right image, cell boundaries visualized via DAPI staining). Replicon #2 encodes both p28 and p88, and GFP in place of p38, thus replicates in most of treated cells to produce green fluorescence (middle right image). **(C)** Cells co-infected by replicons #1 and 2. Rescue of replicon #1 in a fraction of cells led to mCherry but not GFP fluorescence, indicating that p28 expressed from the replicating #1 blocks #2, even though the former had to rely on p88 provided by the latter. Note that the constructs were provided in the form of cDNA, thus replicon #2 could be transcribed and translated to provide p88 independent of replication.

Considering the highly specific nature of SIE-mediated blockage of superinfectors, it was previously proposed the sequence-specific RNA silencing might be the underlying mechanism (Baulcombe, 2004). RNA silencing could still play an important role in SIE of some viruses (Ratcliff et al., 1999; Kurihara and Watanabe, 2003). However, its participation in SIE of other RNA viruses has been cast in doubt by multiple recent studies (Ziebell et al., 2007; Zhang et al., 2015; Zhang and Qu, 2016). Notably, our recent investigations using turnip crinkle virus (TCV) as an SIE model revealed a protein-based mechanism (Zhang et al., 2017). TCV is a very simple virus with a single-stranded, positive sense (+) RNA genome of 4,054 nucleotides (nt) that encodes just five proteins (**Figure 1A**). Only 5′ proximally encoded p28, and the larger p88 arising from the translational read-through of the p28 stop codon, are absolutely required for TCV genome replication. Note that both p28 and p88 are translated directly from TCV genomic RNA, whereas other TCV proteins, including the p8 and p9 movement proteins, and the p38 capsid protein (CP)/RNA silencing suppressor, are primarily translated from subgenomic RNAs synthesized in infected cells (**Figure 1A**).

We found that SIE in TCV infections is elicited by p28 of the primary TCV, and it targets p28 encoded by the superinfecting TCV to block the replication of the latter. This conclusion is supported by multiple lines of evidence. First, p28 expressed independent of TCV replication, especially when fused at its C-terminus with an epitope tag (e.g., HA) or a fluorescent protein (e.g., GFP), robustly blocks the replication of a TCV replicon (Zhang et al., 2017). More importantly, p28 expressed in a replication-dependent manner likewise exerted strong SIE on a different TCV replicon (Zhang et al., 2017). This experiment is a little complex, thus warrants a bit more explanation. **Figure 1B** shows two TCV replicons. Replicon #1 had deletions within p88, p8, and p9 coding regions, and harbored an mCherry coding sequence in place of p38. As a result, this replicon encoded p28 as the sole TCV protein, and could not replicate itself to produce mCherry fluorescence (the top right image). Keep in mind that mCherry expression does not occur unless the replicon replicated to produce sgRNA2. Replicon #2 encoded intact p28 and p88, thus replicated efficiently to produce GFP fluorescence (the middle right image). When these two replicons were mixed and co-delivered into the same cells, most cells expressed GFP, but not mCherry, suggesting that in these cells, only replicon #2 replicated, and that its replication did not cause the #1 to become replicated (bottom row, second image). Importantly, approximately 5% cells did express mCherry but not GFP, indicating that in these cells replicon #1 did manage to utilize p88 translated from replicon #2 for its own replication. The most striking fact worth repeating is that in these cells GFP fluorescence was never detected, despite the fact that the GFP-encoding replicon #2 must be present in these cells to provide p88. The compelling take-home message here is that replication-dependent expression of p28 from replicon #1 exerts robust SIE on replicon #2.

The next set of results identifies p28 as the target of SIE. The SIE-eliciting capacity of p28 could be abolished by tagging its N-terminus with a small peptide (G11, the last β-strand of GFP). Two different TCV replicons, both encoding the G11-p28 fusion in place of p28, but different fluorescent proteins (GFP and mCherry) in place of p38, co-replicated in the same cells, indicating that SIE was abolished by replacing p28 with G11-p28 in TCV replicons (Zhang et al., 2017). More importantly, the G11-p28-encoding replicons were unable to replicate when partnered with another replicon encoding the wild-type p28, indicating that wild-type p28 dominantly represses the G11-p28-encoding replicon. Indeed, thanks to the distinct intracellular behavior of p28 and G11-p28, we could observe that G11-p28 became trapped by wild-type p28 (see later).

Additional supportive evidence can be found in the original publication (Zhang et al., 2017). Collectively our investigations demonstrate that at least in TCV, the replication step is the target of SIE, and this particular virus achieves SIE by using a replication protein (p28) to target its own copies translated from the superinfector. Therefore, p28 facilitates the replication of the primary virus, but represses the replication of superinfector, and p28 itself is both the elicitor and target of SIE. It is important to note that SIE elicitors encoded by potyviruses like wheat streak mosaic virus and triticum mosaic virus are proteins that do not appear to have direct roles in viral genome replication (protease and capsid protein; Tatineni and French, 2016). However, these viruses translate nearly all proteins as one single polyprotein that needs to be proteolytically processed to release functional proteins. Consequently, the identified SIE elicitor could achieve SIE by interfering with the processing of the polyprotein, thereby shutting down the replication of the superinfector.

## How Does A Replication Protein Inactivate Its Own Copies Translated From the Superinfector?

We established that p28, a protein needed for TCV replication, is both the elicitor and target of SIE. To remind readers of a key characteristic of SIE, it is worth emphasizing that SIE does not occur unless the primary virus and the superinfector are closely related. Indeed researchers frequently use derivatives of the same virus as primary virus and superinfector to demonstrate SIE (Takahashi et al., 2007; Miyashita and Kishino, 2010; Zhang et al., 2015, 2017). Our recently published study likewise used TCV variants that were modified to express GFP and mCherry, respectively, from a subgenomic RNA (Zhang et al., 2017). Consequently, p28 expressed from the primary TCV is molecularly indistinguishable from p28 translated from the superinfector genome! How can then the former p28 single out the latter p28 for repression?

**FIGURE 2:**
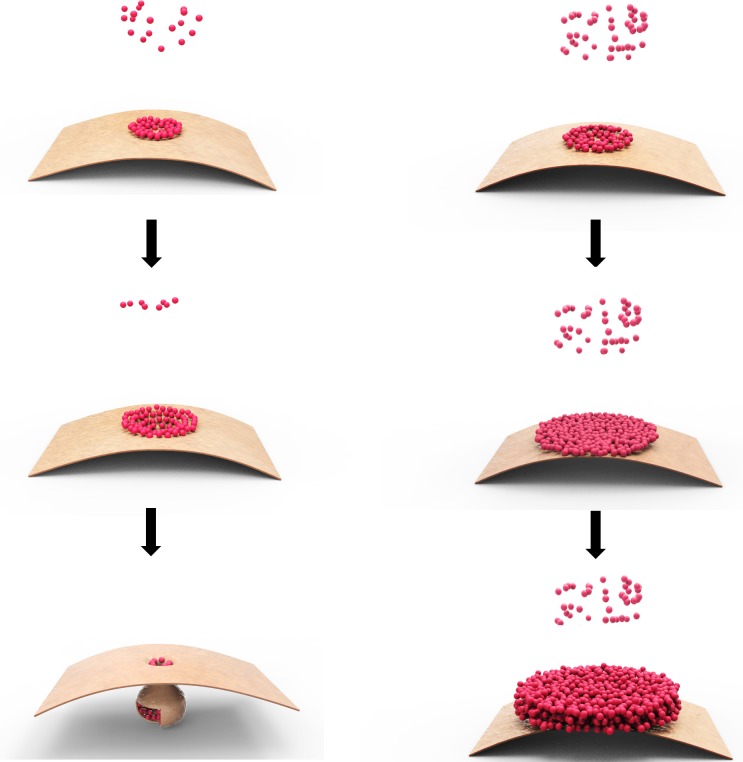
Model of p28 polymerization at different intracellular concentrations. The small dots represent monomeric p28, whereas the bent sheet represents a section of mitochondrial outer membrane. The left column depicts the situation when p28 concentration is relatively low, causing self-limited polymerization of small lattice on the surface of mitochondrial membrane, which eventually becomes enclosed through membrane curving and invagination. The right column depicts the situation when p28 concentration is high; causing the polymerization to proceed swiftly and the lattice expands quickly, making it impossible to become enclosed by mitochondrial membrane. The exposed lattice, or possibly its multi-layered derivatives, could indefinitely recruit new p28 monomers, blocking them from engaging in replication.

The model we invoked is that p28 acts as a replication facilitator at the early stage of TCV infection, when its intracellular concentration is relatively low, but transitions to a repressive state once its concentration rises to a certain threshold, probably as a result of robust p28 translation from newly replicated TCV genomes (**Figure 2**). The molecular basis of this active-to-repressive transition probably lies in the ability of p28 to polymerize. The mechanism of TCV p28 participation in genome replication is likely very similar to the 1a protein encoded by brome mosaic virus (BMV), and p33 of tomato bushy stunt virus (TBSV), which self-interact to form two-dimensional lattices on intracellular membranes (Schwartz et al., 2002, 2004; Xu and Nagy, 2016), causing membrane to curve and invaginate in order to form pear-shaped, membrane-enclosed spherules that become virus replication complexes (VRCs) with the recruitment of viral RNA (Russo and Martelli, 1982; Blake et al., 2007; **Figure 2**, left).

Biochemically, the polymerization of p28, and analogous proteins encoded by other viruses (e.g., TBSV p33 and BMV 1a), is probably slow and self-contained when their intracellular concentration is low, at the beginning of the replication cycle. It is self-contained because polymerization inevitably lowers the concentration of monomeric copies in the surrounding environment, making it harder for the polymer core to recruit more monomers. The self-containment of polymerization is probably also important in that it permits the enclosure of the polymeric lattice by the anchoring membrane to form spherules.

Extending from this same biochemical property, polymerization of p28 is likely greatly accelerated once large quantities of new TCV genomes are produced through replication, and become templates for p28 translation. The acceleration of p28 polymerization, even absent of any conformational changes of p28 monomers, is likely to cause the polymeric lattices to quickly expand in size, making it impossible to be enclosed by membranes, thus incapable of executing its replication function (**Figure 2**, right). More importantly, the unenclosed polymers would be free to recruit even more monomers, thus not only perpetuating the replicationally inactive state, but also denying superinfectors the chance to replicate by sequestering the p28 (and probably RNA as well; see later).

This model is consistent with our observation that C-terminally tagged, highly SIE-inducing p28 variants (p28-GFP and p28-mCherry) formed large-sized, irregularly shaped intracellular foci, whereas an N-terminally tagged, SIE-defective but replication-competent p28 variant (G11-p28, visualized through co-expression of the complementing G1-10 protein) distributes diffusely inside the cells (Zhang et al., 2017). Furthermore, when G11-p28 (plus G1-10) was co-expressed with a tag-free p28, the diffused distribution of G11-p28 was converted into large intracellular foci in approximately 50% of the cells (Zhang et al., 2017). This result not only provided indirect evidence for the formation of large-sized polymers by tag-free p28, but also suggested that such polymers, once formed, could trap G11-p28 monomers and convert them into the polymeric state. Because similar foci formed by p28-GFP/mCherry correlated with a strong repression of TCV replication, the fact that tag-free p28 could also form this type of structures is consistent with the idea that these foci represent the repressive state of p28 responsible for eliciting SIE.

## Are Superinfecting Viruses the “Intended” Targets of SIE?

Here the quote by Richard Dawkins, cited at the beginning of this article, becomes particularly relevant. Assuming SIE operates by targeting the replication of superinfectors, and assuming mechanisms of SIE in other viruses are similar to TCV, namely through viral proteins that switch between two different states in a concentration-dependent manner, we ask the obvious question of how SIE singles out superinfectors while leaving the progeny genomes alone, because by definition the superinfectors and progeny genomes are essentially identical. Using the TCV example, how do the polymeric p28 foci manage to capture p28 monomers translated from superinfecting TCV, but steering clear those translated from newly synthesized TCV genomes?

We advocate that SIE most likely cannot distinguish between progeny viruses and superinfectors. Thousands, if not millions, of progenies are typically produced in a single virus-infected cell within a short period of time, vastly outnumbering the superinfectors, making the latter unlikely to be the primary target of SIE. To put it differently, we argue that the progeny viruses must be the intended target of SIE, whereas the superinfectors are the “collateral damage.”

## What Is the Evolutionary Rationale for SIE As A Mechanism to Thwart the Replication of Progeny Viruses?

**FIGURE 3:**
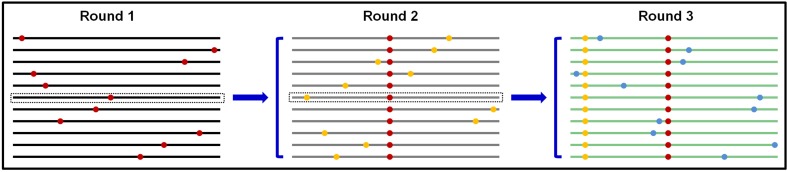
Predicted scenario if progeny TCV genomes are permitted to repeat replication in the same cells. Round 1 replication produces progeny genomes that contain one random error per genome on average. Using the middle genome as an example, permitting it to proceed to round 2 would proliferate the round 1 error (red dot) into more progenies, and compound it with additional random errors (yellow dots). Repetition of this process would result in genomes with too many errors that are predicted to be less fit.

SIE is apparently an adaptive trait for many viruses, especially many of the plant-infecting (+) RNA viruses. The question now is why viruses would favor a function that impedes the replication of their own progenies in a given cell. We reason that by keeping progenies from repeating replication in the cells of their “parents,” all progenies within a given cell would be direct descendants of one or very few founding viruses. This outcome has important implications in balancing genetic diversity of virus populations against the piling up of excessive mutations, many of them expected to be deleterious (**Figure 3**).

The RNA-dependent RNA polymerase (RdRP) encoded by many RNA viruses are known to be error prone, estimated to introduce approximately one error per progeny genome. This error-prone replication is thought to be important for viruses to build a pool of progeny genomes that differ from each other randomly yet minimally, preparing them for adaptation to potentially diverse new hosts. However, should these error-containing genomes be allowed to repeat replication in the cells of founding genome(s), the errors introduced through first round of replication would proliferate into more progenies, and be compounded by additional new errors (**Figure 3**). Such repeatedly replicated genomes, containing more errors, are likely to be progressively less fit, and could be precipitously driven to extinction. Conversely, a mechanism that prevents progeny genomes from re-replicating in the cells of their “parent,” in the form of SIE, is expected to be positively selected during virus evolution in order to ensure all progenies contain a minimal number of random errors. Such a mechanism thus provides a molecular basis for the “stamping machine” replication mode long advocated for RNA viruses (Martinez et al., 2011).

Would these errors not be compounded by new errors in the next set of cells? There is evidence to suggest that viral genomes are subject to quality check prior to embarking on replication to ensure at least certain key functions are preserved before they are replicated (Sun and Qu, unpublished). For instance, errors that disrupt critical *cis*-acting RNA elements would cause the corresponding error-containing genomic RNAs to be excluded from further replication (Newburn and White, 2015; Guo et al., 2017). In addition, recombination likely plays a key role in weeding out deleterious errors by scrambling genome sections from several founding genomes (Xiao et al., 2016).

## “Childless Aunts” Are Not Useless

The repressive effect of SIE is not limited to progeny viruses and superinfectors. It extends to those “sister” viruses that entered the cells more or less simultaneously, yet did not transit to replication soon enough. This was hinted by numerous previous reports showing most cells treated with a mixture of viral variants replicated only one or a few of them (Li and Roossinck, 2004; Takahashi et al., 2007; Miyashita and Kishino, 2010; Zhang et al., 2015, 2017). Yet this point was most dramatically illustrated by a recently published study: cells receiving thousands of variants of the same virus – tomato mosaic virus – supported the replication of fewer than six of them (Miyashita et al., 2015). Thus, an overwhelming majority of the “sister” genomes that entered the cells were excluded from the replication process! How can SIE be established so swiftly? Did these “sister” genomes themselves contribute to the establishment of SIE that excluded them? Do they also act “altruistically” to help the replication of the few “lucky parents”?

Our bold hypothesis that addresses these questions is that most, if not all, of the incoming genomes contribute to the initial pool of replication proteins, p28 and p88 in the case of TCV, even though only a few of them will ever be replicated. While we do not have direct evidence for this, we did observe that a TCV replicon whose replication was blocked by p28-GFP overexpression still synthesized p28 (Zhang et al., 2017). And another TCV replicon repressed by SIE was still able to provide p88 to a different replicon (**Figure 1**). Additionally, an earlier study with a different virus (poliovirus) showed that translation occurred at numerous intracellular sites shortly after virus entry, when (-) RNA was detected at only a few discrete spots, suggesting active translation initiated from multiple virus genomes (Egger and Bienz, 2005).

This arrangement is important not just for theoretical reasons. In real virus infections most cells receive viruses from neighboring cells/tissues, most likely in large numbers, with the possible exception being the cells that encounter viruses first, from external sources (insects, mechanical, etc. See later). An obvious advantage of engaging all of these “sister” genomes in active translation is that a sufficient amount of replication proteins becomes available for constructing VRCs *en masse* within a short time after virus entry. As a result, genome replication in these cells does not have to rely on replication proteins translated from progeny genomes. We further speculate that the level of replication proteins in most cells could even be sufficient for establishing a partial SIE state that is infrequently overcome by a few genomic RNAs, thus accounting for the inability of most viral genomes to replicate (Miyashita et al., 2015). Of course, these “sister” genomes are further blocked from the replication process once replication of the “lucky few” commences and produces progeny genomes that template the translation of even more replication proteins, thus solidifying the SIE state.

The above hypothesis helps address the question of how one or a few actively replicating genomes could possibly induce the enormous amount of VRCs observed in virus-infected cells. For example, both TCV and flock house virus (FHV) establish VRCs, or spherules, on the mitochondrial outer membrane. At least 20 spherules were identifiable in just one mitochondrion section of a TCV-infected turnip cell (Blake et al., 2007). Similarly, at least 30 spherules of different sizes were identifiable in one mitochondrion section of a cell infected by FHV (Ertel et al., 2017). Considering only a thin mitochondrial section was examined, the actual number of spherules per mitochondria could easily exceed 100. The total number of spherules per cell would be well over 2,000 if only 20 mitochondria in each cell house VRCs. Assuming TCV p28 functions in a similar manner as BMV 1a, approximately 100 p28 molecules would be needed for each spherule (Schwartz et al., 2002), making the total number of p28 molecules needed for spherule construction to be at least 200,000 per cell.

This challenge becomes solvable if all or most of the incoming genomes engage in translation of replication proteins that equip a few of them for replication. A recent report estimated that an average mRNA of yeast translates approximately 700 protein molecules (Lahtvee et al., 2017). Assuming this ratio holds true for plant mRNAs, and viral RNAs being three times more efficient than an average cellular mRNA, one TCV genomic RNA could then translate more than 2,000 p28 molecules. As a result, 100 “sister” TCV genomes could be enough to supply the p28 (and p88) needed for building 2,000 spherules.

Finally, we wish to emphasize that the “one cycle” replication we envision does not mean the founding genomes are replicated only once. Rather, they likely act as templates repeatedly to produce multiple direct copies of themselves. This may not be hard to accept for the (-) strand replication intermediates of (+) RNA viruses. What we envision is that (+) RNA genomes could be themselves templates for multiple copies of (-) RNA intermediates. Consistent with this speculation, Sindbis virus, an animal virus with (+) RNA genome, was found to have different requirements for (-) and (+) RNA synthesis, meaning that some VRCs only support (-) RNA synthesis, presumably producing multiple (-) RNA intermediates from a single (+) RNA (Lemm and Rice, 1993; Hellström et al., 2017). This arrangement would make it possible to generate thousands of new progenies from one single founding genome.

## Concluding Remarks

We summarized recently published findings concerning cross protection and SIE, and proposed an integrated model that not only accounts for these new results, but also pivots into a new mechanistic framework for SIE. The central claim of our model is that SIE manifests a virus-encoded, evolutionarily conserved function that minimizes the proliferation of replication errors and, at least with the virus we studied (TCV), this function is realized through a concentration-dependent acceleration of polymerization of a replication protein. While some of our predictions remain to be experimentally validated, the underlying evolutionary rationale of the main thesis gives us confidence that it will likely withstand rigorous interrogations. Below we try to anticipate and address four additional questions related to the model.

First, does the expansive structure responsible for SIE (**Figure 2**, right), formed by p28 polymerization in the case of TCV, contain viral RNA? This question remains to be resolved experimentally but we suspect that it likely does. As a replication protein p28 is expected to help recruit viral RNA to VRCs through protein-RNA interactions. Similar recruitment of RNA could also occur in the SIE-inducing structures, and it could further strengthen the repressive state by stopping viral RNA from entering pre-formed VRCs. It could also provide multiple p28-interacting sites on the same RNA, which could then each nucleate p28 polymerization, causing the stacking of p28 layers and stabilization of the repressive structure.

Second, do the cells that receive only a few viral genomes experience a delay in SIE? We mentioned earlier that most cells in a plant receive viruses from neighboring cells or tissues that already replicated the virus to thousands of copies, thus likely receive hundreds of nearly identical viral genomes. The translation of these genomes probably provides replication proteins needed for the replication of a few of them. However, in any infected plant there are inevitably a few cells that encounter the invading viruses first, probably receiving just a few viruses. Assuming our model holds true, in these cells the replication proteins translated from the incoming genomes are probably insufficient to establish a robust SIE state. As a result, we anticipate that in these cells a fraction of progeny genomes probably will become re-replicated. This outcome would be consistent with the observation that in turnip mosaic virus -infected plant cells a small fraction of cells did undergo geometric replication, even though more than 90% of genomes followed “stamping machine” replication (Martinez et al., 2011). This probably also explains why cultured cells infected with lower multiplicity of infection (MOI) units of viruses could produce progeny viruses that repeat replication in the same cells (Schulte et al., 2015). However, we find it hard to imagine to see repetition of more than two cycles, unless either the virus or the cells contain mutations that compromise SIE.

Our discussions so far exclusively concerned viruses with (+) RNA genomes. How applicable is our model to other viruses? While the mechanistic details could differ, it is interesting to note that the single-stranded (ss) DNA viruses in the family *Geminiviridae* replicate through a rolling circle mode, despite that fact that they rely on the DNA-dependent DNA polymerases of their plant hosts for the replication process. Plant DNA polymerases typically replicate DNA semi-conservatively, copying both DNA strands. Although the ssDNA genomes of geminiviruses are first converted into a double-stranded form, the ensuing replication is nevertheless rolling circle. We speculate that rolling circle replication serves a similar purpose as SIE by ensuring the same parental genome is used as the template for all progenies in the same cell.

Why should we care about the mechanisms of SIE? SIE underlies the cross protection phenomenon in virus-infected plants. The revelation that SIE is controlled by one or a few virus-encoded proteins could allow us engineer attenuated, yet still SIE-potent viruses in order to achieve more consistent cross protection. A mechanistic understanding of SIE could also potentially guide rational design of live vaccines for animal and human viruses. If our prediction is correct, then mutant viruses with SIE disrupted could over-replicate to accumulate many deleterious errors, leading to less invasive viruses that are more readily cleared by the host immune system.

In summary, we propose a model that offers an alternative explanation for SIE. Rigorous testing of predictions arisen from this model could lead to an improved understanding of SIE and virus replication, and provide guidance for novel preventive and therapeutic approaches for virus diseases.

## Author Contributions

Writing-original draft: X-FZ and FQ. Writing-review and editing: FQ, X-FZ, SZ, QG, RS, and TW.

## Conflict of Interest Statement

The authors declare that the research was conducted in the absence of any commercial or financial relationships that could be construed as a potential conflict of interest.
